# New Staphylinidae (Coleoptera) records with new collection data from New Brunswick, Canada: Scaphidiinae, Piestinae, Osorinae, and Oxytelinae

**DOI:** 10.3897/zookeys.186.2506

**Published:** 2012-04-26

**Authors:** Reginald P. Webster, Jon D. Sweeney, Ian DeMerchant

**Affiliations:** 1 Natural Resources Canada, Canadian Forest Service - Atlantic Forestry Centre, 1350 Regent St., P.O. Box 4000, Fredericton, NB, Canada E3B 5P7

**Keywords:** Staphylinidae, Scaphidiinae, Piestinae, *Siagonium*, Osorinae, *Clavispinus*, Oxytelinae, *Mitosynum*, new distributional records, Canada, New Brunswick

## Abstract

Nine species of Scaphidiinae are newly reported for New Brunswick, Canada, bringing the total number of species known from the province to 12. *Scaphium castanipes* Kirby, *Baeocera inexspectata* Löbl and Stephen, *Baeocera securiforma* (Cornell), *Scaphisoma repandum* Casey, and *Toxidium gammaroides* LeConte are reported for the first time from the Maritime provinces. *Siagonum punctatum* LeConte and *Siagonum stacesmithi* Hatch, and the subfamily Piestinae are reported for the first time from New Brunswick. The subfamily Osoriinae is reported for the first time from New Brunswick and the Maritime provinces based on the collection of three species: *Clavilispinus prolixus* (LeConte), *Thoracophorus costalis* (Erichson), and a *Lispinodes* species. The *Lispinodes* species is also newly recorded for Canada. Six species of Oxytelinae are newly recorded from New Brunswick, bringing the total number of species of this subfamily known to the province to 20. *Apocellus sphaericollis* (Say) and *Platystethus americanus* Erichson are new to the Maritime provinces. Additional locality and bionomic data are presented for *Mitosynum vockerothi* Campbell, and the male genitalia are illustrated for the first time. Collection and bionomic data are presented for all included species.

## Introduction

Intensive collecting of Staphylinidae in New Brunswick by the first author since 2003 and records obtained from by-catch samples during a study to develop a general attractant for the detection of invasive species of Cerambycidae have yielded many new provincial records. These are being published in a series of papers, each focusing on one or more subfamilies. This paper treats staphylinids of the subfamilies Scaphidiinae, Piestinae, Osorinae, and Oxytelinae. A brief synopsis of each subfamily is included in the results below.

## Methods and conventions

### Collection methods

A variety of collection methods were employed to collect the species reported in this study. Details are outlined in Campbell (1973) and Webster et al. (2009, Appendix). See Webster et al. (2012) for details of the methods used for deployment of Lindgren 12-funnel traps and sample collection. A description of the habitat was recorded for all specimens collected during this survey. Locality and habitat data are presented exactly as on labels for each record. This information, as well as additional collecting notes, is summarized and discussed in the collection and habitat data section for each species.

### Specimen preparation

Males of some species (all Scaphidiinae) were dissected to confirm their identity. The genital structures were dehydrated in absolute alcohol and mounted in Canada balsam on celluloid microslides and pinned with the specimens from which they originated.

### Distribution

Distribution maps, created using ArcMap and ArcGIS, are presented for each species in New Brunswick. Every species is cited with current distribution in Canada and Alaska, using abbreviations for the state, provinces, and territories. New provincial records are indicated in bold under Distribution in Canada and Alaska. The following abbreviations are used in the text:

**Table:** 

**AK**	Alaska	**MB**	Manitoba
**YT**	Yukon Territory	**ON**	Ontario
**NT**	Northwest Territories	**QC**	Quebec
**NU**	Nunavut	**NB**	New Brunswick
**BC**	British Columbia	**PE**	Prince Edward Island
**AB**	Alberta	**NS**	Nova Scotia
**SK**	Saskatchewan	**NF & LB**	Newfoundland and Labrador*

* Newfoundland and Labrador are each treated separately under the current Distribution in Canada and Alaska.

Acronyms of collections examined and referred to in this study are as follows:

**AFC** Atlantic Forestry Centre, Natural Resources Canada, Canadian Forest Service, Fredericton, New Brunswick

**CNC** Canadian National Collection of Insects, Arachnids and Nematodes, Ottawa, Ontario

**NBM** New Brunswick Museum, Saint John, New Brunswick

**RWC** Reginald P. Webster Collection, Charters Settlement, New Brunswick

## Species accounts

All records below are species newly recorded for New Brunswick, Canada, unless noted otherwise (additional records). Species followed by ** are newly recorded from the Maritime provinces (New Brunswick, Nova Scotia, Prince Edward Island) of Canada; species followed by *** are newly recorded from Canada. A list of species of Scaphidiinae, Piestinae, Osoriinae, and Oxytelinae currently known from New Brunswick is given in Table 1.

The classification of the Scaphidiinae, Piestinae, Osorinae, and Oxytelinae follows Bouchard et al. (2011).

**Table 1.** Species of Scaphidiinae, Piestinae, Osoriinae, and Oxytelinae (Staphylinidae) recorded from New Brunswick, Canada.

**Family Staphylinidae Latreille**
**Subfamily Scaphidiinae Latreille**
**Tribe Scaphidiini Latreille**
*Scaphidium quadriguttatum* Say*
**Tribe Scaphiini Achard**
*Scaphium castanipes* Kirby**
**Tribe Scaphisomatini Casey**
*Baeocera apicalis* LeConte
*Baeocera deflexa* Casey
*Baeocera indistincta* Löbl and Stephan
*Baeocera inexspectata* Löbl and Stephan**
*Baeocera securiforma* (Cornell)**
*Baeocera youngi* (Cornell)*
*Scaphisoma convexum* Say*
*Scaphisoma repandum* Casey**
*Scaphisoma rubens* Casey*
*Toxidium gammaroides* LeConte**
**Subfamily Piestinae Erichson**
*Siagonum punctatum* LeConte*
*Siagonium stacesmithi* Hatch**
**Subfamily Osoriinae Erichson**
**Tribe Thoracophorini Reitter**
*Clavilispinus prolixus* (LeConte)**
*Lispinodes* sp.***
*Thoracophorus costalis* (Erichson)**
**Subfamily Oxytelinae Fleming**
**Tribe Euphaniini Reitter**
*Deleaster dichrous* (Gravenhorst)
*Mitosynum vockerothi* Campbell
*Syntomium grahami* Hatch
**Tribe Coprophilini Heer**
*Coprophilus castoris* Campbell
*Coprophilus striatulus* (Fabricius)*
**Tribe Blediini Ádám**
*Bledius annularis* LeConte
*Bledius basalis* LeConte
*Bledius neglectus* Casey
*Bledius nitidicollis* LeConte
*Bledius philadelphicus* Fall
*Bledius politus* Erichson
*Bledius tau* LeConte
**Tribe Oxytelini Fleming**
*Carpelimus obesus* (Kiesenwetter)
*Anotylus rugosus* (Fabricius)
*Anotylus insecatus* (Gravenhorst)*
*Anotylus tetracarinatus* (Block)*
*Apocellus sphaericollis* (Say)**
*Oxytelus sculptus* Gravenhorst*
*Oxytelus laqueatus* (Marsham)
*Platystethus americanus* Erichson**

**Notes:** *New to province; **New to Maritime provinces; ***New to Canada.

### Family Staphylinidae Latreille, 1802

**Subfamily Scaphiini Achard, 1924**

Cornell (1967) and Löbl and Stephan (1993) reviewed the *Baeocera* of North America. Leschen et al. (1990) provided a review and keys of the nine species of *Scaphisoma* from the Ozark Highland (in Oklahoma, Arkansas, and Missouri). However, the genera Scaphidium and Scaphisoma are in need of revision. Species in this subfamily are mycophagous on many fungi species, including polypore fungi and on slime molds (Newton 1984; Leschen 1988; Newton et al. 2001; Brunke et al. 2011). Adults inhabit decaying wood, fungi, and leaf litter and also occur under bark and in compost. Campbell (1991) reported no species of Scaphidiinae for New Brunswick. Later, Löbl and Stephan (1993) in their review of the *Baeocera* of North America reported *Baeocera apicalis* LeConte, *Baeocera indistincta* Löbl and Stephan, and *Baeocera deflexa* Casey from New Brunswick. Here, we report nine species of Scaphidiinae new to the province (Table 1).

### Tribe Scaphidiinae Latreille, 1806

#### Scaphidium quadriguttatum

Say, 1823

http://species-id.net/wiki/Scaphidium_quadriguttatum

Map 1

##### Material examined. 

**New Brunswick, Queens Co.**, Cranberry Lake P.N.A. (Protected Natural Area), 46.1125°N, 65.6075°W, 22–29.VI.2009, M. Roy & V. Webster, old red oak forest, Lindgren funnel trap (1 ♂, RWC). **Restigouche, Co.**, Dionne Brook P.N.A., 47.9064°N, 68.3441°W, 27.VI-14.VII.2011, M. Roy & V. Webster, old-growth white spruce and balsam fir forest, Lindgren funnel trap (1, NBM). **York Co.**, Charters Settlement, 45.8406°N, 66.7321°W, 8.VI.2003, R. P. Webster, mixed forest, on foliage (1, RWC); 14 km WSW of Tracy, S of Rt. 645, 45.6741°N, 66.8661°W, 10-26.V.2010, R. Webster & C. MacKay, old mixed forest with red and white spruce, red and white pine, balsam fir, eastern white cedar, red maple, and *Populus* sp., Lindgren funnel trap (1, AFC).

**Map 1.**
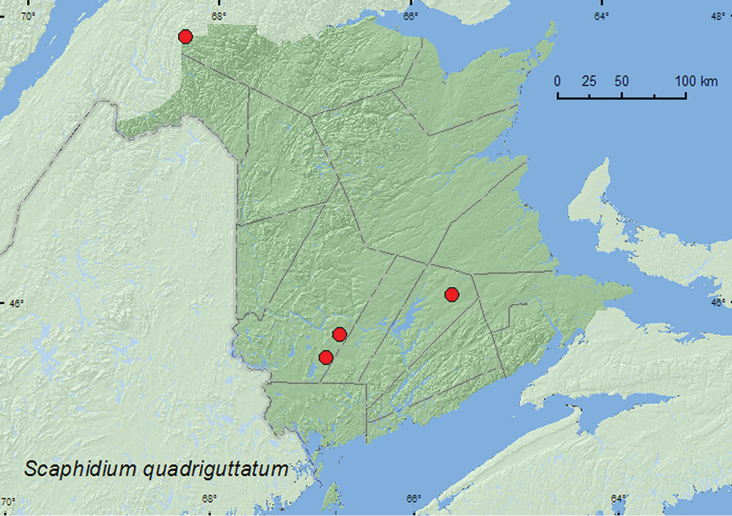
Collection localities in New Brunswick, Canada of *Scaphidium quadriguttatum*.

##### Collection and habitat data.

*Scaphidium* spp. are associated with old logs and polypore fungi (Newton et al. 2000). In New Brunswick, one individual of S. *quadriguttatum* was collected from foliage (beating) in a mixed forest, and others were captured in Lindgren funnel traps deployed in an old (180-year-old trees) mixed forest, an old red oak (*Quercus rubra* L.) forest, and an old-growth balsam fir (*Abies balsamsea* (L.) Mill.) and white spruce (*Picea glauca* (Moench) Voss) forest. Adults were collected during May, June, and July.

##### Distribution in Canada and Alaska.

ON, QC, **NB**, NS (Campbell 1991; Bishop et al. 2009).

### Tribe Scaphidiini Latreille, 1806

#### Scaphium castanipes

Kirby, 1837**

http://species-id.net/wiki/Scaphium_castanipes

Map 2

##### Material examined. 

**New Brunswick, Restigouche Co.** Mount Carleton Provincial Park, Mount Sagamook, 625 m elev., 47.4112°N, 66.8599°W, 2.IX.2006, R. P. Webster, mixed forest, on decaying gilled mushroom (1, RWC); Dionne Brook P.N.A., 47.9064°N, 68.3441°W, 28.VII.2011, R. P. Webster, old-growth white spruce and balsam fir forest, in gilled mushrooms (4, RWC).

**Map 2.**
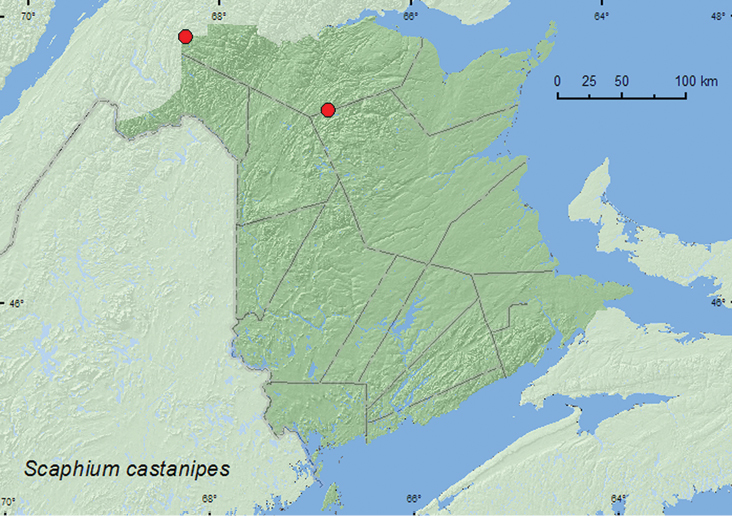
Collection localities in New Brunswick, Canada of *Scaphium castanipes*.

##### Collection and habitat data.

The larvae of this species feed on mushrooms (Ashe 1984). The New Brunswick specimens were collected from a decaying gilled mushroom on a mountain side (625 m elev.) and from gilled mushrooms in an old-growth white spruce and balsam fir forest. Adults were collected during June and August.

##### Distribution in Canada and Alaska.

AK, YK, NT, BC, AB, MB, ON, QC, **NB** (Campbell 1991).

### Tribe Scaphisomatini Casey, 1893

#### Baeocera inexspectata

Löbl and Stephan, 1993**

http://species-id.net/wiki/Baeocera_inexspectata

Map 3

##### Material examined. 

**New Brunswick, Charlotte Co.**, S of Little Pocologan River, 45.1546°N, 66.6254°W, 7.V.2007, R. P. Webster, mature eastern white cedar swamp/forest, in moss and leaf litter (1 ♂, RWC). **Sunbury Co.**, Acadia Research Forest, 46.0188°N, 66.3765°W, 18.VI.2007, R. P. Webster, mature red spruce and red maple forest, sifting leaf litter and moss (2 ♂, RWC).

**Map 3.**
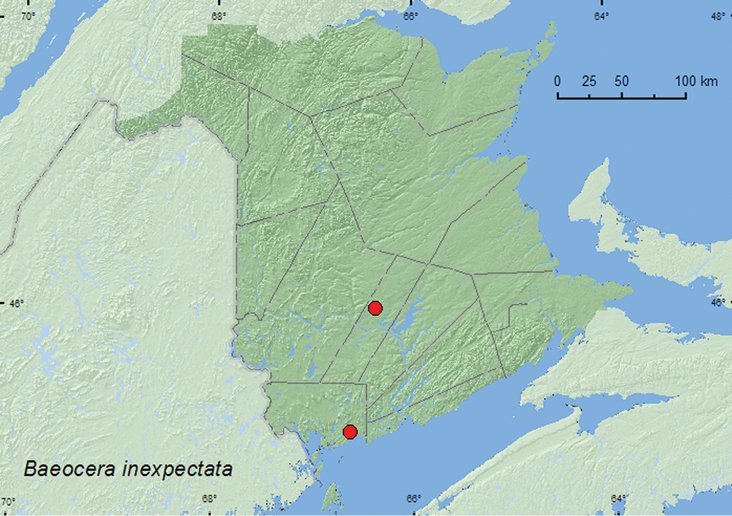
Collection localities in New Brunswick, Canada of *Baeocera inexspectata*.

##### Collection and habitat data.

*Baeocera inexspectata* adults were sifted from moss and leaf litter in a mature eastern white cedar (*Thuja occidentalis* L.) swamp/forest and in a mature red spruce (*Picea rubens* Sarg.) and red maple (*Acer rubrum* L.) forest. Adults were captured during May and June. Nothing was previously known about the habitat associations of this species.

##### Distribution in Canada and Alaska.

SK, **NB** (Löbl and Stephan 1993). Additional sampling in appropriate habitats will probably show this species occurs in intervening areas between New Brunswick and Saskatchewan.

#### Baeocera securiforma

(Cornell, 1967)**

http://species-id.net/wiki/Baeocera_securiforma

Map 4

##### Material examined. 

**New Brunswick, Queens Co.**, Upper Gagetown, bog adjacent to Hwy 2, 45.8316°N, 66.2346°W, 12.IV.2006, R. P. Webster, tamarack bog, in sphagnum hummock and litter on bog margin (1 ♂, RWC); Rees, near Grand Lake, 46.0016°N, 65.9466°W, 29.V.2007, S. Makepeace & R. Webster, in nest contents of barred owl in artificial nest box (1 ♂, RWC). **Restigouche Co.**, near MacFarlane Brook, 47.6018°N, 67.6263°W, 25.V.2007, R. P. Webster, old growth eastern white cedar swamp, in moss (2 ♂, RWC).

**Map 4.**
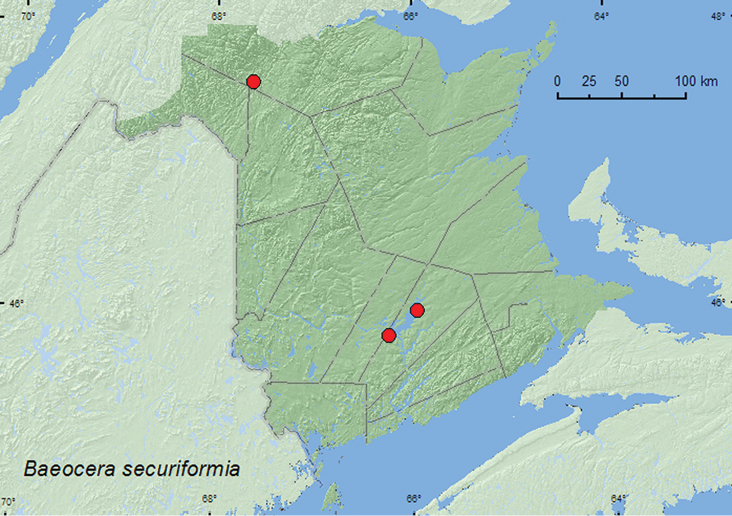
Collection localities in New Brunswick, Canada of *Baeocera securiforma*.

##### Collection and habitat data.

Löbl and Stephan (1993) reported that *Baeocera securiforma* occurred in similar habitats as *Baeocera congener* Casey, namely in a variety of forest litter. The New Brunswick specimens were collected from moss in an eastern white cedar swamp, in litter and sphagnum in a sphagnum hummock on the margin of a *Carex* marsh and a tamarack (*Larix laricina* (Du Roi) K. Koch) bog, and from the nest contents of a barred owl (*Strix varia* Barton). The adults were collected during April and May.

##### Distribution in Canada and Alaska.

MB, ON, QC, **NB** (Löbl and Stephan 1993).

#### Baeocera youngi

(Cornell, 1967)

http://species-id.net/wiki/Baeocera_youngi

Map 5

##### Material examined. 

**New Brunswick, Queens Co.**, Cranberry Lake P.N.A., 46.1125°N, 65.6075°W, 11–18.VI.2009, R. Webster & M.-A. Giguère, old red oak forest, Lindgren funnel trap (1 ♂, RWC).

**Map 5.**
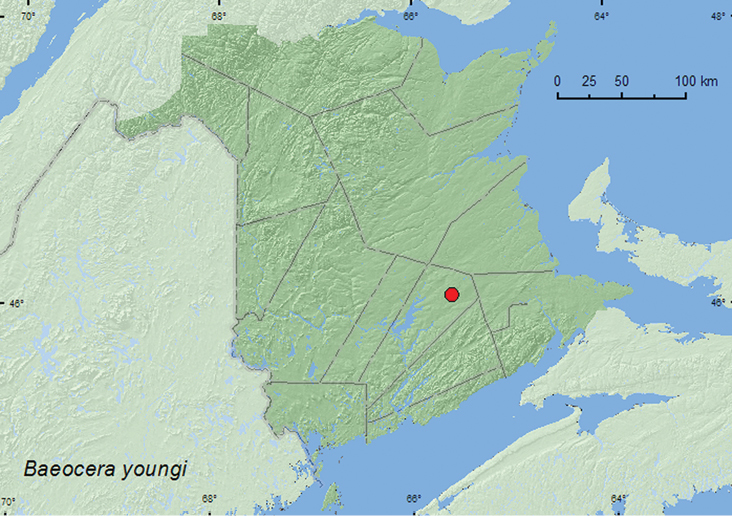
Collection localities in New Brunswick, Canada of *Baeocera youngi*.

##### Collection and habitat data.

Löbl and Stephan (1993) reported this species from moist hardwood litter. The specimen from New Brunswick was captured during June in a Lindgren funnel trap deployed in an old red oak forest.

##### Distribution in Canada and Alaska.

SK, MB, ON. QC, **NB**, NS (Löbl and Stephan 1993).

#### Scaphisoma convexum

Say, 1825

http://species-id.net/wiki/Scaphisoma_convexum

Map 6

##### Material examined. 

**New Brunswick, Carleton Co.**, Meduxnekeag Valley Nature Preserve, 46.1907°N, 67.6740°W, 6.VII.2006, 12.IX.2008, R. P. Webster, hardwood forest, on gilled mushroom (1 ♂, 1 ♀, RWC). **Restigouche, Co.**, Dionne Brook P.N.A., 47.9064°N, 68.3441°W, 27.VI–14.VII.2011, M. Roy & V. Webster, old-growth white spruce and balsam fir forest, Lindgren funnel traps (1 ♀, RWC). **Sunbury Co.**, Maugerville, Portobello Creek N.W.A., 45.9031°N, 66.4268°W, 11.IX.2006, R. P. Webster, red oak and red maple forest, on stalked polypore mushroom on forest floor (3 ♂, RWC).

**Map 6.**
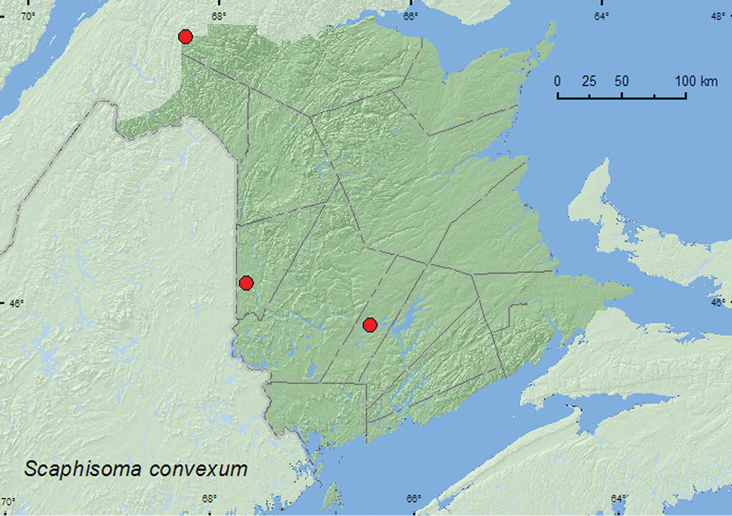
Collection localities in New Brunswick, Canada of *Scaphisoma convexum*.

##### Collection and habitat data.

*Scaphisoma convexum* was reported from a variety of Agaricales and Polyporales fungi in the Ozark Highland and was reared from the polypore *Tyromyces* (Leschen et al. 1990). In New Brunswick, this species was collected from gilled mushrooms and from stalked polypore mushrooms on the forest floor. Adults were found in hardwood forests. One individual was captured in a Lindgren funnel trap in an old-growth white spruce and balsam fir forest. This species was collected during June, July, and September.

##### Distribution in Canada and Alaska.

MB, ON, QC, **NB** (Campbell 1991).

#### Scaphisoma repandum

Casey, 1894

http://species-id.net/wiki/Scaphisoma_repandum

Map 7

##### Material examined. 

**New Brunswick, Carleton Co.**, near Hovey Hill P.N.A., 46.1152°N, 67.7632°W, 10.V.2005, R. P. Webster, mixed forest with cedar, vernal pond margin, in moist leaf litter on muddy soil (1, RWC). **Sunbury Co.**, Sheffield, Portobello Creek N.W.A., 45.8950°N, 66.2725°W, 12.V.2004, silver maple forest (swamp), in leaf litter (1, RWC). **York Co.** Charters Settlement, 45.8342°N, 66.7450°W, 10.VI.2004, R.P. Webster, mixed forest, wood pile, under bark (3, RWC).

**Map 7.**
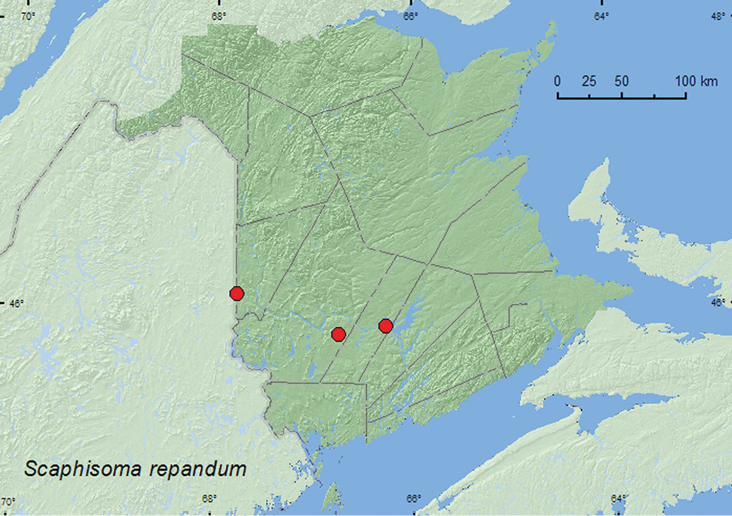
Collection localities in New Brunswick, Canada of *Scaphisoma repandum*.

##### Collection and habitat data.

In New Brunswick, *Scaphisoma repandum* was collected from moist leaf litter on a vernal pond margin in a mixed forest, in moist leaves in a silver maple (*Acer saccharinum* L.) swamp and under loose bark of wood in a wood pile in a mixed forest. Adults were collected during May and June.

##### Distribution in Canada and Alaska.

ON, **NB** (Campbell 1991).

#### Scaphisoma rubens

Casey, 1894

http://species-id.net/wiki/Scaphisoma_rubens

Map 8

##### Material examined. 

**New Brunswick, Albert Co.**, Caledonia Gorge P.N.A., 45.8257°N, 64.7791°W, 6.VII.2011, R. P. Webster, old hardwood forest (sugar maple and beech), on *Polyporus varius* (1, NBM); same locality and collector but 45.8175°N, 64.7770°W, 6.VII.2011, old hardwood forest (sugar maple and beech), under bark of sugar maple (1, NBM). **Carleton Co.**, Meduxnekeag Valley Nature Preserve, 46.1940°N, 67.6801°W, 12.VIII.2004, R. P. Webster, hardwood forest, in fleshy fungi in various stages of decay (2, RWC); Jackson Falls, Bell Forest, 46.2200°N, 67.7231°W, 18.VIII.2006, R. P. Webster, mature hardwood forest, in fleshy polypore fungi on dead standing beech (1 ♂, RWC); same locality, forest type and collector, 12–19.VI.2008, Lindgren funnel trap (1, AFC); same locality and forest type, 16–21.VI.2009, 14–19.VII.2009, R. Webster & M.-A. Giguère, Lindgren funnel traps (2, AFC). **Charlotte Co.**, 10 km NW of New River Beach, 45.2110°N, 66.6170°W, 15–29.VI.2010, R. Webster & C. MacKay, old growth eastern white cedar forest, Lindgren funnel trap (1, AFC). **Queens Co.**, Cranberry Lake P.N.A, 46.1125°N, 65.6075°W, 2.IX.2009, R. P. Webster, old red oak forest, in small stalked polypore fungus on forest floor (1, AFC). **Restigouche Co.**, Jacquet River Gorge P.N.A., 47.8201°N, 65.9992°W, 12.VIII.2010, R. P. Webster, black spruce, balsam fir & old eastern white cedar forest, in decaying mushrooms (1, NBM); Dionne Brook P.N.A., 47.9064°N, 68.3441°W, 31.V–15.VI.2011, M. Roy & V. Webster, old-growth white spruce and balsam fir forest, Lindgren funnel trap (1, NBM). **Saint John Co.**, Dipper Harbour, 45.1176°N, 66.3806°W, 12.IX.2006, R. P. Webster, red spruce forest, on gilled mushroom (2 ♂, RWC). **Sunbury Co.**, Maugerville, Portobello Creek N.W.A., 45.9031°N, 66.4268°W, 11.IX.2006, R. P. Webster, red oak and red maple forest, on stalked polypore mushroom on forest floor (1 ♂, RWC); Acadia Research Forest, 46.0173°N, 66.3741°W, 17.VII.2007, R. P. Webster, 8.5 year-old regenerating mixed forest, in gilled mushroom on stump (1, AFC); same locality data, forest type, and collector, 14.V.2007, sifting leaf litter (1, AFC); same locality and collector but 45.9799°N, 66.3394°W, 18.VII.2007, 17.VIII.2007, 18.IX.2007, mature red spruce and red maple forest, in gilled mushrooms (2, AFC). **York Co.** Canterbury, Browns Mountain Fen, 45.8965°N, 67.6344°W, 5.VIII.2004, R. P. Webster, mixed forest, in decaying fleshy fungi (1, RWC); Charters Settlement, 45.8395°N, 66.7391°W, 1.VIII.2004, R. P. Webster, mixed forest, u.v. light (1, RWC); same locality but 45.8286°N, 66.7365°W, 15.VIII.2004, old red spruce and cedar forest, in decaying mushrooms (1, RWC); 15 km W of Tracy off Rt. 645, 45.6848°N, 66.8821°W, 25.V–1.VI.2009, R. Webster & M.-A. Giguère, old red pine forest, Lindgren funnel trap (1, AFC).

**Map 8.**
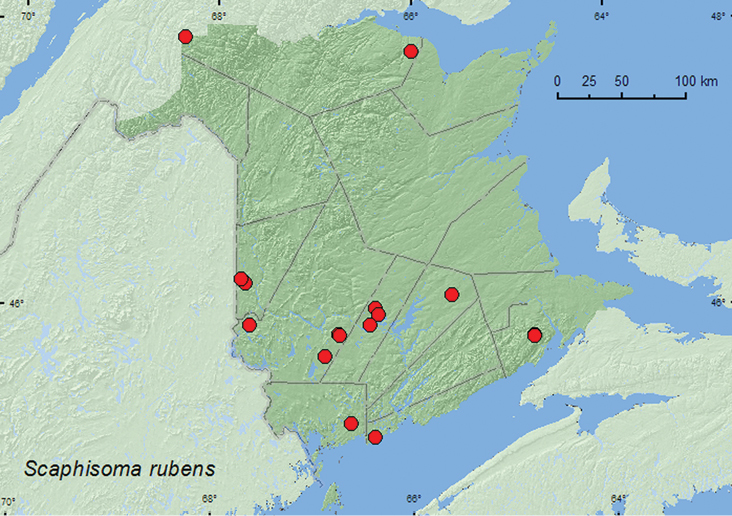
Collection localities in New Brunswick, Canada of *Scaphisoma rubens*.

##### Collection and habitat data.

*Scaphisoma rubens* was found in a variety of forest types in New Brunswick. These included mature hardwood forests (sugar maple and American beech (*Fagus grandifolia* Ehrh.)), mixed forests, a regenerating mixed forest, old eastern white cedar forests, an old red oak forest, a red oak and red maple forest, an old red pine (*Pinus resinosa* Ait.) forest, a mature red spruce and red maple forest, an old-growth white spruce and balsam fir forest, and a conifer forest with black spruce (*Picea mariana* (Mill.) B.S.P.), balsam fir, and eastern white cedar. Adults were taken from fleshy fungi, gilled mushrooms, decaying fleshy fungi, decaying mushrooms, fleshy polypore fungi on dead standing American beech, small stalked polypore fungi on forest floor, *Polyporus varius* Fr. on rotten logs and standing dead sugar maples, under bark of sugar maple, and from leaf litter. Several adults were captured in Lindgren funnel traps. Adults were collected during May, June, July, August, and September.

##### Distribution in Canada and Alaska.

QC, **NB**, NS (Campbell 1991, Dollin et al. 2008).

#### Toxidium gammaroides

LeConte, 1860**

http://species-id.net/wiki/Toxidium_gammaroides

Map 9

##### Material examined. 

**New Brunswick, Carleton Co.**, Meduxnekeag Valley Nature Preserve, 46.1896°N, 67.6700°W, 26.IX.2007, R. P. Webster, hardwood forest, on *Pholiota* sp. on base of dead standing beech (1 ♂, RWC); Jackson Falls, Bell Forest, 46.2200°N, 67.7231°W, 1–8.VI.2009, 19–31.VII.2009, M.-A. Giguère & R. Webster, mature hardwood forest, Lindgren funnel traps (2, RWC). **York Co.**, (Canterbury) near Browns Mountain, 45.8874°N, 66.6274°W, 8.IX.2007, R. P. Webster, hardwood forest, in polypore fungi under bark (1, RWC); Charters Settlement, 45.8286°N, 66.7365°W, 13–17.VII.2008, R. P. Webster, mature mixed forest, Lindgren funnel trap (1, RWC).

**Map 9.**
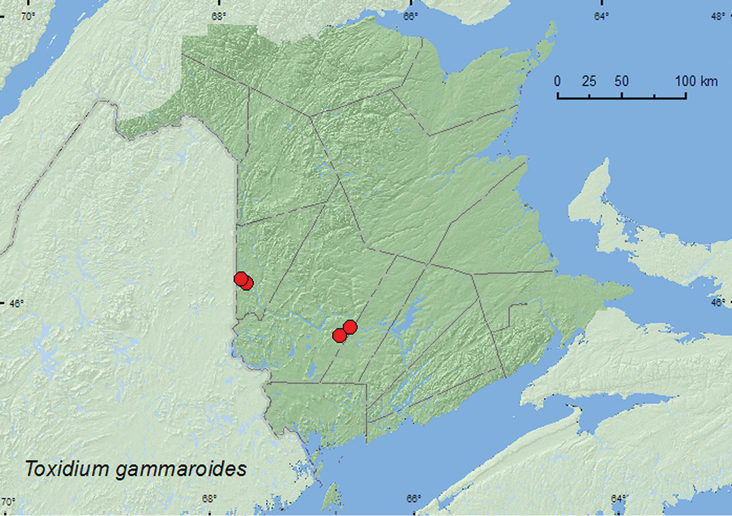
Collection localities in New Brunswick, Canada of *Toxidium gammaroides*.

##### Collection and habitat data.

Members of this genus are associated with polypore species on old logs (Newton et al. 2001). In New Brunswick,*Toxidium gammaroides* was found in mature hardwood forests and in a mixed forest. Adults were collected from a group of *Pholiota* sp. on the base of a dead standing American beech and in polypore fungi under bark. Adults were also captured in Lindgren funnel traps. This species was collected during June, July, and September.

##### Distribution in Canada and Alaska.

ON, QC, **NB** (Campbell 1991).

### Subfamily Piestinae Erichson, 1839

In Canada, the subfamily Piestinae is represented by the genus *Siagonium* with three species (See Moore (1975) for key to species). Members of this genus occur under bark of dead trees, but very little is known about their biology (Brunke et al. 2011). Here, we report *Siagonium stacesmithi* Hatch, *Siagonium punctatum* LeConte, and this subfamily for the first time from New Brunswick.

#### Siagonium punctatum

LeConte, 1866

http://species-id.net/wiki/Siagonium_punctatum

Map 10

##### Material examined. 

**New Brunswick, Albert Co.**, Caledonia Gorge P.N.A. near Turtle Creek, 45.8380°N, 64.8484°W, 6.VII.2011, R. P. Webster, old-growth hardwood forest (sugar maple and yellow birch), under bark of sugar maple log (1, NBM). **Carleton Co.**, Meduxnekeag Valley Nature Preserve, 46.1907°N, 67.6740°W, 7.VI.2007, R. P. Webster, hardwood forest, under bark of sugar maple (2, RWC); Jackson Falls, Bell Forest, 46.2210°N, 67.7210°W, 26.VI.2007, R. P. Webster, mature hardwood forest, u.v. light (1, RWC). **Charlotte Co.**, 10 km NW of New River Beach, 45.2110°N, 66.6170°W, 16-26.VII.2010, R. Webster & V. Webster, old growth eastern white cedar forest, Lindgren funnel trap (1, AFC). **Queens Co.**, Cranberry Lake P.N.A, 46.1125°N, 65.6075°W, 10–15.VII.2009, R. Webster & M.-A. Giguère, old red oak forest, Lindgren funnel trap (1 ♂, RWC); same locality data and forest type, 13–25.V.2011, 7–22.VI.2011, M. Roy & V. Webster, Lindgren funnel traps (3, RWC). **York Co.**, Charters Settlement, 45.8395°N, 66.7391°W, 23–27.V.2009, R. P. Webster, mature mixed forest, Lindgren funnel trap (1, RWC); 15 km W of Tracy off Rt. 645, 45.6848°N, 66.8821°W, 19–25.V.2009, R. Webster & M.-A. Giguère, old red pine forest, Lindgren funnel trap (1, RWC); same locality data and forest type, 8-20.VI.2011, M. Roy & V. Webster, Lindgren funnel trap (1, RWC); 14 km WSW of Tracy, S of Rt. 645, 45.6741°N, 66.8661°W, 26.IV-10.V.2010, R. Webster & C. MacKay, old mixed forest with red and white spruce, red and white pine, balsam fir, eastern white cedar, red maple, and *Populus* sp., Lindgren funnel trap (1, RWC).

**Map 10.**
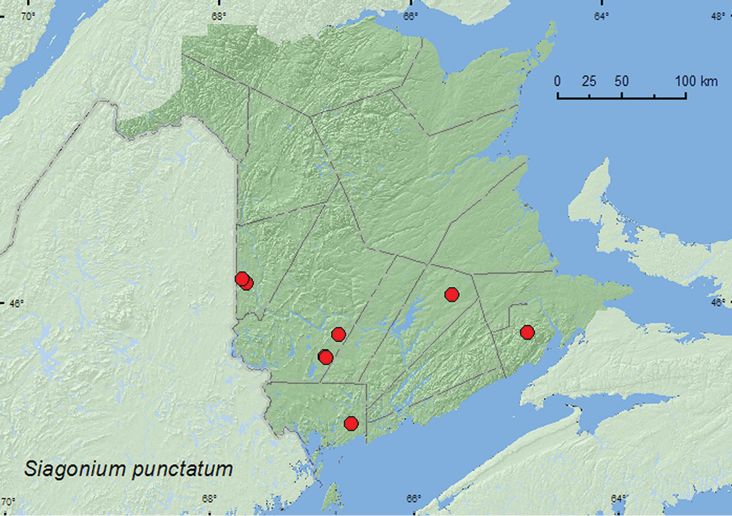
Collection localities in New Brunswick, Canada of *Siagonium punctatum*.

##### Collection and habitat data.

Members of this genus occur under bark of dead trees and sometimes at light (Brunke et al. 2011). In New Brunswick, this species was captured in Lindgren funnel traps deployed in an old-growth eastern white cedar forest, an old red oak forest, an old-growth northern hardwood forest (sugar maple and yellow birch (*Betula alleghaniensis* Britt.)), an old red pine forest, and an old mixed forest. Adults were also collected from under tight bark of sugar maple and at an ultraviolet light in hardwood forests. Adults were captured during April, May, June, and July.

##### Distribution in Canada and Alaska.

ON, QC, **NB**, NS (Campbell and Davies 1991; Dollin et al. 2008).

#### Siagonium stacesmithi

Hatch, 1957**

http://species-id.net/wiki/Siagonium_stacesmithi

Map 11

##### Material examined. 

**New Brunswick, Restigouche Co.**, Dionne Brook P.N.A., 47.9030°N, 68.3503°W, 30.V-15.VI.2011, M. Roy & V. Webster, old-growth northern hardwood forest, Lindgren funnel trap (1, RWC).

**Map 11.**
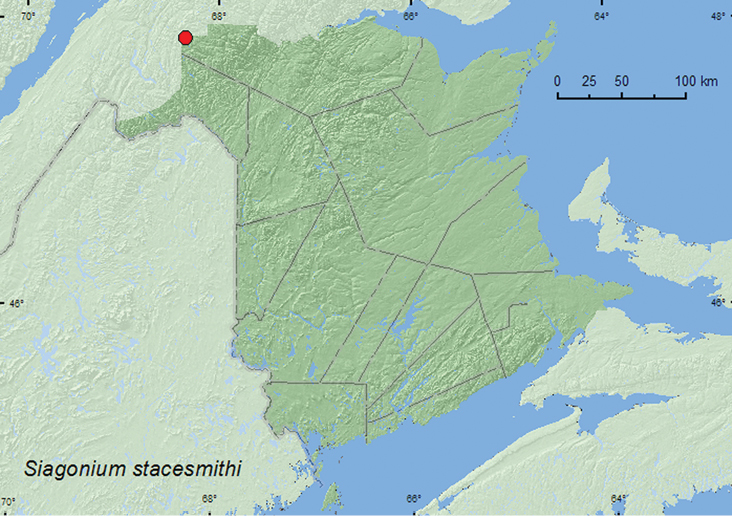
Collection localities in New Brunswick, Canada of *Siagonium stacesmithi*.

##### Collection and habitat data.

The specimen from New Brunswick was captured during June in a Lindgren funnel trap deployed in an old-growth northern hardwood forest with sugar maple and yellow birch. Hatch (1957) reported this species in the West from under bark of ponderosa pine (*Pinus ponderosa* Douglas ex Lawson & C. Lawson), on newly cut wood after sundown, and taken during evening flight.

##### Distribution in Canada and Alaska.

YT,BC, AB, SK, MB, ON, QC, **NB** (Hatch 1957). Distribution is based on Hatch’s (1957) types of *Siagonium stacesmithi* and specimens in the CNC.

### Subfamily Osoriinae Erichson, 1839

In Canada, the Osoriinae is represented by three genera, *Clavilispinus*, *Thoracophorus*, and *Renardia*, with six species (Campbell and Davies 1991). Representatives of all four genera occur in eastern Canada. Members of this subfamily are taxonomically poorly known, and little is known about their biology (Brunke et al. 2011). Species from eastern Canada have been found under bark, in leaf litter, and in ant nests in decaying wood and are probably saprophagous or mycophagous (Newton et al. 2000; Brunke et al. 2011). Most members of this subfamily appear to be rare in eastern Canada (Brunke et al. 2011). Campbell and Davies (1991) did not report any members of this subfamily for New Brunswick or the Maritime provinces. Here, we report *Clavilispinus prolixus* (LeConte), *Thoracophorus costalis* (Erichson), and a *Lispinodes* species, which is a new genus for Canada (Table 1).

### Tribe Thoracophorini Reitter, 1909

#### Clavilispinus prolixus

(LeConte, 1877)**

http://species-id.net/wiki/Clavilispinus_prolixus

Map 12

##### Material examined. 

**New Brunswick, Charlotte Co.**, 10 km NW of New River Beach, 45.2110°N, 66.6170°W, 30.IV-17.V.2010, R. Webster & V. Webster, old growth eastern white cedar forest, Lindgren funnel trap (1, RWC). **Queens Co.**, Cranberry Lake P.N.A, 46.1125°N, 65.6075°W, 25.V–7.VI.2011, 7–22.VI.2011, 7–13.VII.2011, M. Roy & V. Webster, mature (old) red oak forest, Lindgren funnel traps (1, AFC, 1, NBM, 7, RWC).

**Map 12.**
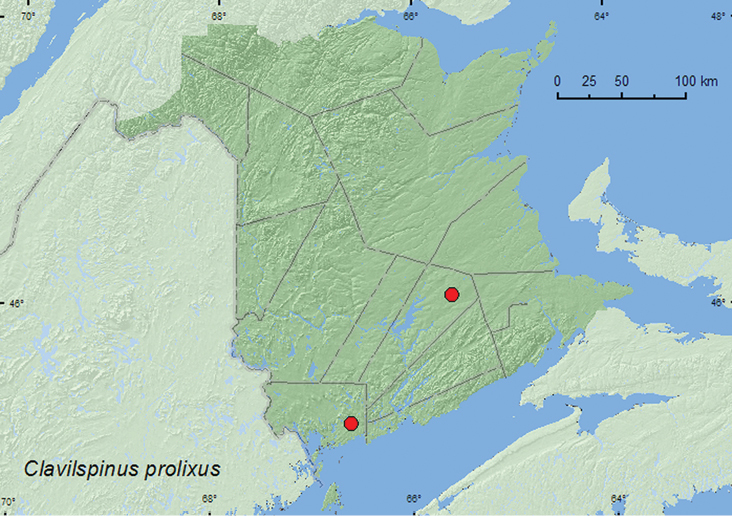
Collection localities in New Brunswick, Canada of *Clavilispinus prolixus*.

##### Collection and habitat data.

Some members of this genus are found under bark or in ant nests (*Formica* and *Camponotus*) in rotting logs (Newton et al. 2001). Specimens from New Brunswick were captured in Lindgren funnel traps deployed in an old eastern white cedar forest/swamp and an old red oak forest. Adults were captured during May, June, and July.

##### Distribution in Canada and Alaska.

MB, QC, **NB** (Campbell and Davies 1991).

#### Lispinodes undescribed species ***

Map 13

##### Material examined. Canada, 

**New Brunswick, Queens Co.**, Grand Lake Meadows P.N.A., 45.8227°N, 66.1209°W, 15–29.VI.2010, 29.VI-12.VII.2010, R. Webster, M. Laity, R. Johns, & C. MacKay, old silver maple forest with green ash and seasonally flooded marsh, Lindgren funnel traps (14, AFC, RWC).

**Map 13.**
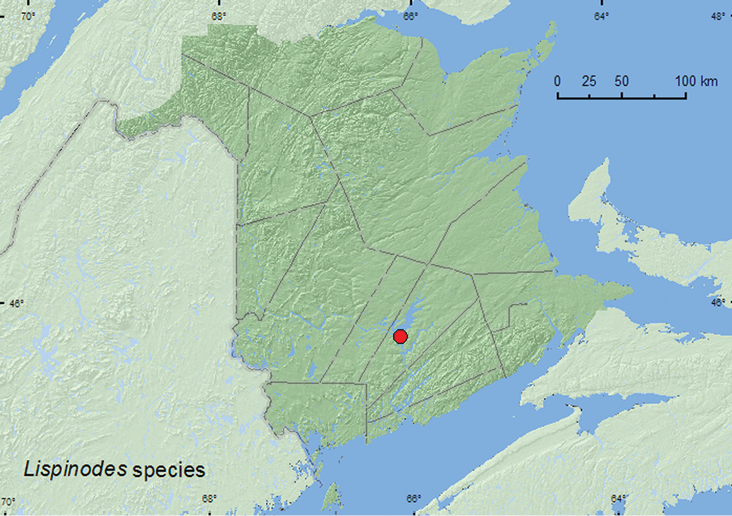
Collection localities in New Brunswick, Canada of *Lispinodes* sp.

##### Collection and habitat data.

Adults of this species were reported from leaf litter (Newton et al. (2000), otherwise little is known about the biology of this species. The New Brunswick specimens were captured in Lindgren funnel traps deployed in an old silver maple swamp. Adults were collected during June and July.

##### Comment.

This is probably the same undescribed species that was reported by Newton et al. (2000) from Michigan and Illinois.

##### Distribution in Canada and Alaska.

**NB** (First Canadian record of this genus).

#### Thoracophorus costalis

(Erichson, 1840)**

http://species-id.net/wiki/Thoracophorus_costalis

Map 14

##### Material examined. 

**New Brunswick, Queens Co.**, Cranberry Lake P.N.A, 46.1125°N, 65.6075°W, 11-18.VI.2009, 1-10.VII.2009, 10-15.VII.2009, 15-21.VII.2009, 21–28.VII.2009, 14–19.VIII.2009, R. Webster & M.-A. Giguère, mature (old) red oak forest, Lindgren funnel traps (19, AFC, RWC); same locality data and forest type, 7–22.VI.2011, M. Roy & V. Webster, Lindgren funnel traps (5, AFC, NBM); Grand Lake Meadows P.N.A., 45.8227°N, 66.1209°W, 5–19.VII.2011, M. Roy & V. Webster, old silver maple forest and seasonally flooded marsh, Lindgren funnel trap (1, NBM). **York Co.**, 15 km W of Tracy off Rt. 645, 45.6848°N, 66.8821°W, 13–17.VII.2008, R .P. Webster, old red pine forest, Lindgren funnel trap (1, RWC); same locality data and forest type, 8–20.VI.2011, M. Roy & V. Webster, Lindgren funnel traps (2, NBM, RWC).

**Map 14.**
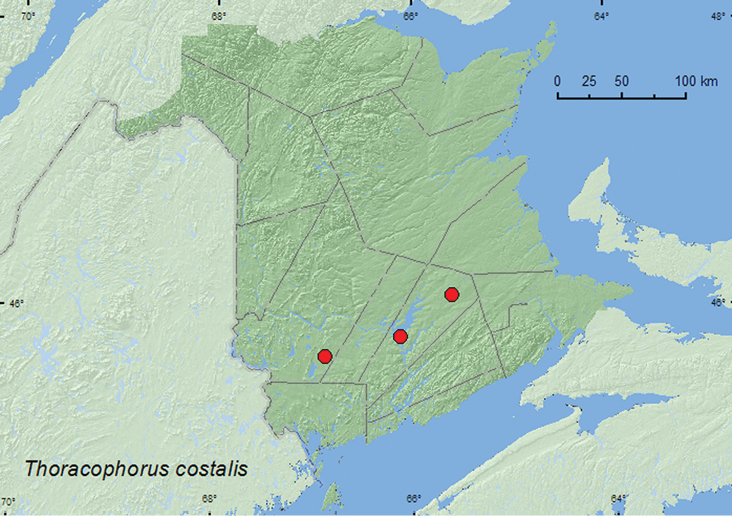
Collection localities in New Brunswick, Canada of *Thoracophorus costalis*.

##### Collection and habitat data.

Brunke et al. (2011) reported this species from under bark, especially large beech logs, and occasionally in leaf litter. In New Brunswick, adults were captured in Lindgren funnel traps deployed in an old red oak forest, an old silver maple swamp, and an old red pine forest. Adults were captured during June, July, and August.

##### Distribution in Canada and Alaska.

MB, ON, QC, **NB** (Campbell and Davies 1991).

### Subfamily Oxytelinae Fleming, 1821

Members of this subfamily occur in a variety of habitats. The Blediini (*Bledius* species) live in tunnels along sun-exposed, sparsely vegetated, freshwater and marine shorelines and feed on algae (Herman 1986). Many members of the Oxytelinae in eastern Canada are usually associated with decaying organic matter, leaf litter, and moss (Brunke et al. 2011). Other species occur along river, stream, and pond margins and in litter. Members of this subfamily are predators, algivores, coprophages, omnivores, or saprophages (Brunke et al. 2011). The *Bledius* were reviewed by Herman (1972, 1976, 1983, 1986), but some genera of Oxtyelinae occurring in eastern Canada, such as the large genus *Carpelimus*, are poorly known and in need of revision.

Nine species of Oxytelinae were reported as occurring in New Brunswick by Campbell and Davies (1991). Klimaszewski et al. (2005) added *Syntomium grahami* Hatch, *Carpelimus obesus* (Kiesenwetter), and *Oxytelus laqueatus* (Marsham). *Deleaster dichrous* (Gravenhorst) was added by Majka and Klimaszewski (2008a) and *Bledius basalis* LeConte by Majka and Klimaszewski (2008c). Here, we report six additional species of Oxytelinae for New Brunswick, bringing the total number of species known from the province to 20 (Table 1).

### Tribe Euphaniini Reitter, 1909

#### Mitosynum vockerothi

Campbell, 1982

http://species-id.net/wiki/Mitosynum_vockerothi

Map 15 Figures 1 2

##### Material examined. 

**Additional New Brunswick records, Charlotte Co.**, near New River, 45.21176°N, 66.61790°W, 2.VI.2006, 7.VII.2006, 7.V.2007, R. P. Webster, small pond/marsh, sifting sphagnum and *Polytrichum commune* on hummock near margin of pond (1 ♂, 8 sex undetermined, RWC). **Sunbury Co.**, Acadia Research Forest, 45.9816°N, 66.3374°W, 17.VIII.2007, R. P. Webster, 8.5 year-old regenerating mixed forest, in sphagnum and leaf litter at bottom of old tire depression (1, AFC).

**Map 15.**
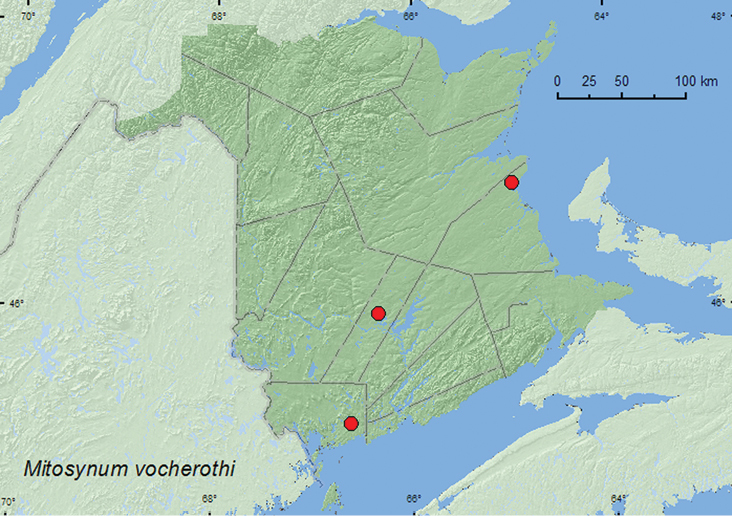
Collection localities in New Brunswick, Canada of *Mitosynum vockerothi*.

**Figure 1.**
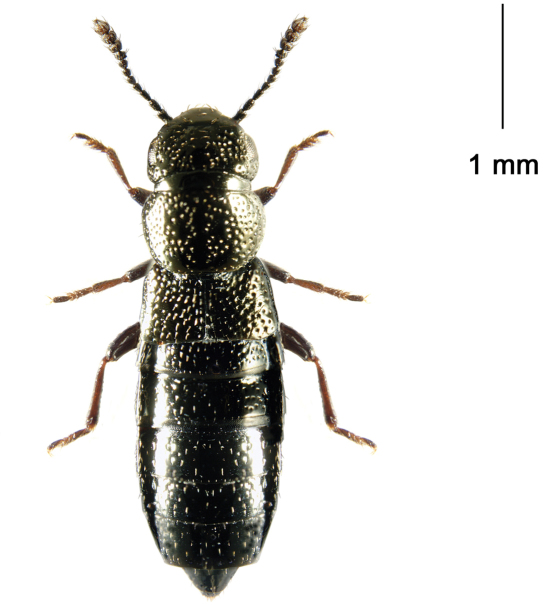
Adult *Mitosynum vockerothi*. Scale = 1 mm

**Figure 2.**
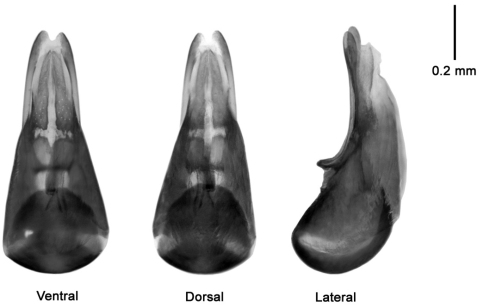
Median lobe of aedeagus *Mitosynum vockerothi*; dorsal, ventral and lateral view.* *

##### Collection and habitat data.

The only previously known adults from the type series of *Mitosynum vockerothi* fromKouchibouguac National Park, New Brunswick were collected from pan traps set at the edge of a sphagnum bog (Campbell 1982). Campbell (1982) suggested that this species, which has reduced eyes and wings, might live in deep layers of leaf litter or in clumps of moss. The recently collected adults of this species were sifted from a large sphagnum and *Polytrichum commune* Hedw. (common haircap moss) hummock near the margin of a small pond and from a layer of sphagnum and leaf litter in the bottom of a deep old tire depression in an 8.5-year-old regenerating mixed forest, supporting Campbell’s suggested habitat association. Adults were collected during June, July, and August.

##### Comments.

*Mitosynum vockerothi* was described from two female specimens (Campbell 1982). Here, we provide an illustration of the dorsal habitus (Fig. 1) and illustrate the male genitalia of this species for the first time (Fig. 2).

##### Distribution in Canada and Alaska.

NB (Campbell and Davies 1991).

### Tribe Coprophilini Heer, 1839

#### Coprophilus castoris

Campbell, 1979

http://species-id.net/wiki/Coprophilus_castoris

Map 16

##### Material examined. 

**Additional New Brunswick records, Albert Co.**, Caledonia Gorge P.N.A. at Caledonia Creek, 45.7935°N, 64.7760°W, 1.VII.2011, R. P. Webster, shaded, rocky, cold, clear brook, splashing gravel (1, RWC). **Restigouche Co.**, Jacquet River Gorge P.N.A., 47.8257°N, 66.0779°W, 24.V.2010, R. P. Webster, partially shaded cobblestone bar near outflow of brook into Jacquet River, under cobblestones and gravel on sand (1, RWC). **York Co.**, Charters Settlement, 45.8395°N, 66.7391°W, 23.IV.2008, R. P. Webster, mature mixed forest, in flight, collected with aerial net between 15:00 and 18:00 h (1, RWC).

**Map 16.**
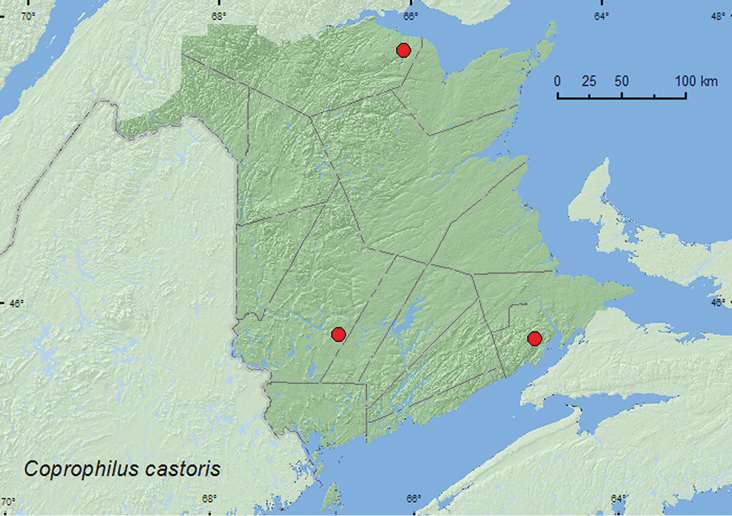
Collection localities in New Brunswick, Canada of *Coprophilus castoris*.

##### Collection and habitat notes.

*Coprophilus castoris* was reported from inside beaver (*Castor canadensis* Kuhl) lodges and collected during an evening flight (St. Andrews, N.B.) (Campbell 1979). The recent New Brunswick specimens of this rare species were found among cobblestones and gravel on sand on a partially shaded cobblestone bar near the outflow of a brook into a river, and among gravel in a cold-shaded brook, and were collected with an aerial net during a late afternoon (15:00–18:00 h) flight. Adults were collected during April, May, and July.

##### Distribution in Canada and Alaska.

ON, QC, NB (Campbell and Davies 1991).

#### Coprophilus striatulus

(Fabricius, 1792)

http://species-id.net/wiki/Coprophilus_striatulus

Map 17

##### Material examined. 

**New Brunswick, Carleton Co.**, Meduxnekeag Valley Nature Preserve, 46.1931°N, 67.6825°W, 31.V.2005, M.-A. Giguère & R. Webster, river margin, under drift material (2, NBM, RWC); Jackson Falls, Bell Forest, 46.2200°N, 67.7231°W, 4–12.VI.2008, R. P. Webster, mature hardwood forest, Lindgren funnel trap (1, AFC). **Restigouche Co.**, Little Tobique River near Red Brook, 47.4465°N, 67.0689°W, 13.VI.2006, R. P. Webster, river margin, under debris on sand clay mix (1, RWC). **York Co.**, Charters Settlement, 45.8395°N, 66.7391°W, 20.IV.2004, 14.V.2005, 23.IV.2006, 14.V.2006, 27.IV.2008, R. P. Webster, mixed forest, in compost (decaying vegetables) (5, NBM, RWC); same locality data, forest type and collector, 27.VIII.2008, in decaying (moldy) corncobs and cornhusks (1, RWC); same locality data, forest type and collector, 23.IV.2008, 6.V.2008, in flight, collected with aerial net between 15:00 and 18:00 h (4, RWC); Canterbury, near Browns Mountain Fen, 45.8977°N, 67.6335°W, 1.VI.2005, R. Webster & M.-A. Giguère, mixed forest, in flight along forest trail (1, NBM).

**Map 17.**
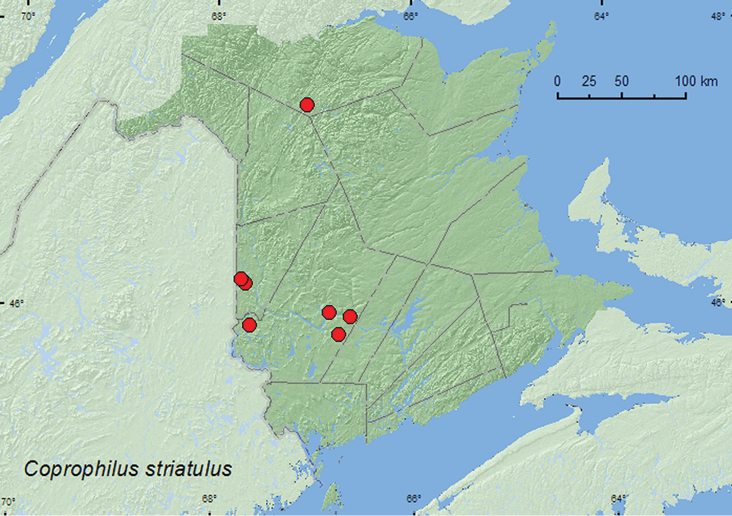
Collection localities in New Brunswick, Canada of *Coprophilus striatulus*.

##### Collection and habitat data.

This adventive species is often found in decaying plant material, decaying vegetables, cow dung, and decaying leaves (Hoebeke 1995). In New Brunswick, this species was collected from under drift material along river margins, in compost (decaying vegetables), and among decaying corncobs and cornhusks. Adults were also collected in flight with an aerial net during a late afternoon (15:00–18:00 h) flight near a mixed forest and along a trail in a mixed forest. One adult was captured in a Lindgren funnel trap in a mature hardwood forest. Adults were captured during April, May, June, and August.

##### Distribution in Canada and Alaska.

ON, QC, **NB**, NS (Hoebeke 1995; Majka and Klimaszewski 2008a).

### Tribe Oxytelini Fleming, 1821

#### Anotylus insecatus

(Gravenhorst, 1806)

http://species-id.net/wiki/Anotylus_insecatus

Map 18

##### Material examined. 

**New Brunswick, Carleton Co.**, Jackson Falls, 46.2257°N, 67.7420°W, 22.V.2010, R. P. Webster, river margin, in gravel on gravel bar (1, RWC). **York Co.**, Fredericton at Saint John River, 45.9588°N, 66.6254°W, 7.VI.2005, R. P. Webster, margin of river, in flood debris (1 ♀, RWC); Keswick River at Rt. 105, 45.9938°N, 66.8344°W, 3.VI.2008, R. P. Webster, upper river margin, in flood debris on sand clay mix (1 ♂, RWC).

**Map 18.**
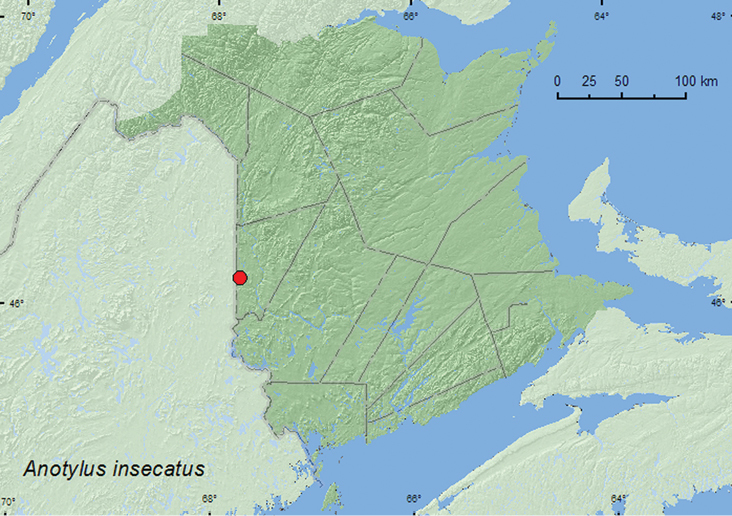
Collection localities in New Brunswick, Canada of *Anotylus insecatus*.

##### Collection and habitat data.

*Anotylus insecatus*is probably a predator of Diptera larvae in bulbs of onions, tulips, and radishes (Campbell and Tomlin 1983; Majka and Klimaszewski 2008b). This adventive species has also been found at sap flows and in decaying plant debris (Campbell and Tomlin 1983) but may also be saprophagous or a scavenger (Hammond 1976). The New Brunswick specimens were found along river margins in flood debris or in gravel. Adults were captured during May and June.

##### Distribution in Canada and Alaska.

AB, SK, MB, ON, QC, **NB**, NS (Campbell and Davies 1991; Majka and Klimaszewski 2008b). Distribution is based on Campbell and Davies (1991), Majka and Klimaszewski (2008b) and specimens from AB, SK, and MB in the CNC (Anthony Davies, personal communication).

#### Anotylus tetracarinatus

(Block, 1799)

http://species-id.net/wiki/Anotylus_tetracarinatus

Map 19

##### Material examined. 

**New Brunswick, Carleton Co.**, Meduxnekeag Valley Nature Preserve, 46.1931°N, 67.6825°W, 8.VI.2005, R. P. Webster, hardwood forest (flood plain forest with butternut), under dog scat (1, RWC); same locality and forest type but 20.VI.2005, M.-A. Giguère & R. Webster, entrance to animal den, in dung (2, RWC). **York Co.**, Douglas, Keswick River at Rt. 105, 45.9922°N, 66.8326W, 9.V.2006, R. P. Webster, upper river margin, in deer dung on sandy soil (1, RWC).

**Map 19.**
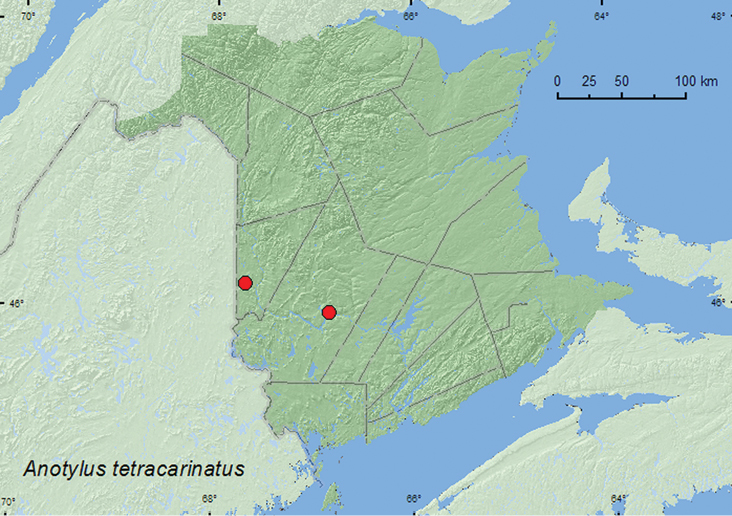
Collection localities in New Brunswick, Canada of *Anotylus tetracarinatus*.

##### Collection and habitat data.

This adventive species occurs in dung, mammal nests, and decomposing fungi (Herman 2001). In New Brunswick, this species was found under dog scat, in dung in an entrance to an animal den, and under deer dung. Adults were captured during May and June.

##### Distribution in Canada and Alaska.

BC, ON, QC, **NB**, NS (Campbell and Davies 1991; Majka and Klimaszewski 2008b). Distribution is based on Campbell and Davies (1991), Majka and Klimaszewski (2008b) and specimens from ON in the CNC (Anthony Davies, personal communication).

#### Apocellus sphaericollis

(Say)**

http://species-id.net/wiki/Apocellus_sphaericollis

Map 20

##### Material examined. 

**New Brunswick, New Brunswick, Albert Co.**, Caledonia Gorge P.N.A. at Crooked Creek, 45.7930°N, 64.7764°W, 1.VII.2011, R. P. Webster, sun-exposed, rocky, cold, clear stream, in drift material (1, NBM). **Madawaska Co.**, Loon Lake, 236 m elev., 47.7839°N, 68.3943°W, 21.VI.2010, R. P. Webster, boreal forest, small lake surrounded by sedges, treading sedges and grasses (1, RWC). **York Co.**, Charters Settlement, 45.8395°N, 66.7391°W, 24.X.2005, 20.IX.2007, 30.VI.2008, R. P. Webster, residential lawn, on bare soil among lawn grass (9, RWC).

**Map 20.**
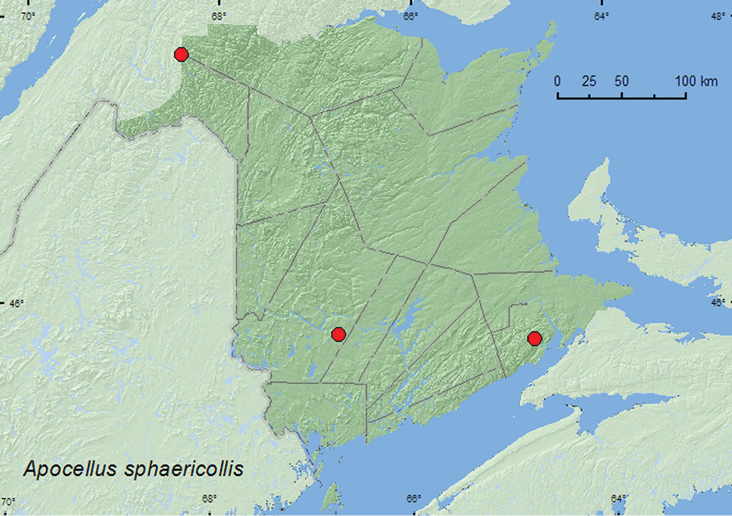
Collection localities in New Brunswick, Canada of *Apocellus sphaericollis*.

##### Collection and habitat data.

*Apocellus* has been found along streams near moss and in open grassy areas (Brunke et al. 2011). Most adults of *Anotylus sphaericollis* (Say)from New Brunswick were collected on bare soil among lawn grasses. One individual was collected by treading sedges and grasses on the margin of a small lake and another from drift material (tree bud material) along a cold sun-exposed stream. Adults were captured during June, July, September, and October.

##### Distribution in Canada and Alaska.

AB, MB, ON, QC, **NB** (Campbell and Davies 1991).

#### Oxytelus sculptus

Gravenhorst, 1806

http://species-id.net/wiki/Oxytelus_sculptus

Map 21

##### Material examined. 

**New Brunswick, York Co.**, Charters Settlement, 45.8395°N, 66.7391°W, 21.VI.2004, 16.X.2004, 10.VII.2005, 27.VIII.2005, 6.IX.2005, 16.IX.2005, 26.IX.2005, 28.IX.2005, R. P. Webster, mixed forest, in compost (decaying vegetables) (1 ♂, 1 ♀, 8 sex undetermined, NBM, RWC); same locality data, forest type, and collector but 29.VI.2005, u.v. light (1 ♂, RWC).

**Map 21.**
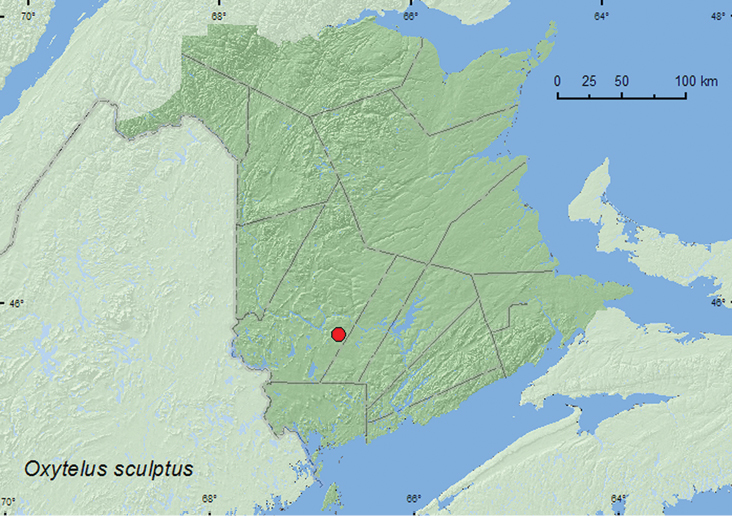
Collection localities in New Brunswick, Canada of *Oxytelus sculptus*.

##### Collection and habitat data.

This adventive species occurs in compost and manure of cattle, horses, and poultry. Most adults from New Brunswick were collected from compost (decaying vegetables). One individual was captured at an ultraviolet light. Adults were collected during June, July, August, September, and October.

##### Distribution in Canada and Alaska.

BC, MB, ON, QC, **NB**, NS (Campbell and Davies 1991; Majka and Klimaszewski 2008b). There are specimens of this species from MB (NIS lot (1994) determined by Anthony Davies (Anthony Davies, personal communication).

#### Platystethus americanus

Erichson**

http://species-id.net/wiki/Platystethus_americanus

Map 22

##### Material examined. 

**New Brunswick, York Co.**, Charters Settlement, 45.8430°N, 66.7275°W, 25.IX.2004, 6.X.2005, R. P. Webster, regenerating mixed forest, baited with pile of decaying mushrooms (2, RWC); same locality and collector but 45.8395°N, 66.7391°W, 23.IV.2008, mixed forest, in flight, collected with aerial net between 15:00 and 18:00 h (1, RWC).

**Map 22.**
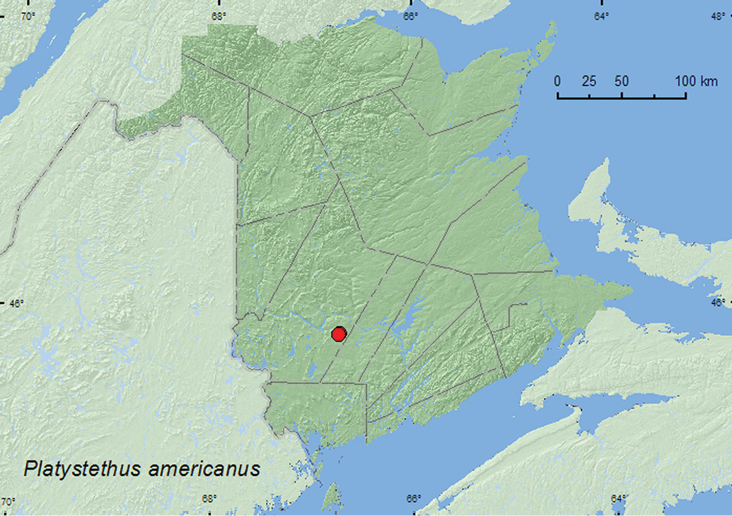
Collection localities in New Brunswick, Canada of *Platystethus americanu*

##### Collection and habitat data.

Newton et al. (2001) reported this species as common in cattle dung. In New Brunswick, adults of this species were sifted from decaying mushrooms and collected with an aerial net during a late afternoon flight. Adults were collected during April and September.

##### Distribution in Canada and Alaska.

BC, AB, SK, MB, ON, QC, **NB** (Campbell and Davies 1991).

## Supplementary Material

XML Treatment for Scaphidium quadriguttatum

XML Treatment for Scaphium castanipes

XML Treatment for Baeocera inexspectata

XML Treatment for Baeocera securiforma

XML Treatment for Baeocera youngi

XML Treatment for Scaphisoma convexum

XML Treatment for Scaphisoma repandum

XML Treatment for Scaphisoma rubens

XML Treatment for Toxidium gammaroides

XML Treatment for Siagonium punctatum

XML Treatment for Siagonium stacesmithi

XML Treatment for Clavilispinus prolixus

XML Treatment for Lispinodes

XML Treatment for Thoracophorus costalis

XML Treatment for Mitosynum vockerothi

XML Treatment for Coprophilus castoris

XML Treatment for Coprophilus striatulus

XML Treatment for Anotylus insecatus

XML Treatment for Anotylus tetracarinatus

XML Treatment for Apocellus sphaericollis

XML Treatment for Oxytelus sculptus

XML Treatment for Platystethus americanus
